# Multi-label Learning for Predicting the Activities of Antimicrobial Peptides

**DOI:** 10.1038/s41598-017-01986-9

**Published:** 2017-05-19

**Authors:** Pu Wang, Ruiquan Ge, Liming Liu, Xuan Xiao, Ye Li, Yunpeng Cai

**Affiliations:** 1 Shenzhen Institutes of Advanced Technology, and Key Lab for Health Informatics, Chinese Academy of Sciences, Shenzhen, Guangdong 518055 China; 2 Shenzhen College of Advanced Technology, University of Chinese Academy of Sciences, Shenzhen, 518055 China; 3 Computer Department, Jingdezhen Ceramic Institute, Jingdezhen, 333403 China; 4 College of Mathematics and Statistics, Shenzhen University, Shenzhen, 518060 China; 5 Present Address: School of Computer Science and Technology, Hangzhou Dianzi University, Hangzhou, Zhejiang, 310018 China

## Abstract

Antimicrobial peptides (AMPs) are peptide antibiotics with a broad spectrum of antimicrobial activities. Activity prediction of AMPs from their amino acid sequences is of great therapeutic importance but imposes challenges on prediction methods due to label interactions. In this paper we propose a novel multi-label learning model to address this problem. A weighted K-nearest neighbor classifier is adopted for efficient representation learning of the sequence data. A multiple linear regression model is then employed to learn a mapping from the classifier score vectors to the target labels, with label correlations considered. Several popular multi-label learning algorithms and feature extraction methods were tested on a comprehensive, up-to-date AMP dataset with twelve biological activities covered and its filtered version with five activities covered. The experimental results showed that our proposed method has competitive performance with previous works and could be used as a powerful engine for activity prediction of AMPs.

## Introduction

With an increasing number of drug-resistant microorganisms, the development of new-generation antibiotics turns into an urgent challenge^1^. Antimicrobial peptides (AMPs) are a potential therapeutic alternative, which are commonly found in the innate immune systems of nearly all kinds of life^2^. These peptides are broad spectrum antibiotics which have been demonstrated to kill bacteria, viruses, fungi and even cancer cells^3^. In addition to antimicrobial, natural AMPs also possess many other activities that are of therapeutic importance, such as wound healing, antioxidant and immune modulation^4^. In recent years, many machine learning methods have been applied in AMP analysis, which may become useful tools to speed up the classification and design of AMPs. However, most existing works only focused on the problem of identifying AMPs from peptide sequences (binary classification problem), or giving them one of several activities (multi-class classification problem)^5–12^. The activity prediction of AMPs is in fact a multi-label learning problem because any AMP may be relevant to one or more activities. In 2013 a two level classifier iAMP-2L was proposed, in which the first level was to identify an AMP, and then the second level involved predicting the activities of AMPs^13^. In this work, however, only five activities were considered and no existing multi-label learning methods were tested. To address these problems, firstly we constructed a new dataset with twelve biological activities covered based on the latest antimicrobial peptide database (APD) and a filtered dataset with only five activities covered but more biological significance; secondly, several previously described multi-label learning methods and an original method presented in this work were tested with both datasets.

As a trending topic in machine learning, multi-label learning is attracting more and more interest. Many multi-label learning algorithms have been proposed and applied in various fields. The easiest way to implement multi-label learning is the Binary Relevance (BR) method^14–16^, which decomposes a multi-label learning problem into multiple binary classification problems for each label respectively, and then all the classical binary classification methods could be used here. The main drawback is that the label correlation is ignored completely, which has been shown to produce negative impacts on classification quality by many previous literature^17^. Label Powerset (LP) is another way to transform the multi-label learning into the traditional multi-class classification by treating each possible label combination as a new class label^18^. There are two limits to the application of LP. Firstly, if a label set does not appear in the training dataset, it will not be predicted; secondly, if there are many candidate labels, then there will be abundant newly mapped classes, and the sample size may be too small for some new classes. RAkEL is an improvement version of LP by splitting the initial label set into small random subsets, and then employs LP method to build classifiers for each subset^19^. Based on the label distribution in K-nearest neighbors, MLKNN uses maximum a posteriori (MAP) to predict the label set of a query sample^20^. Rank-SVM is an adaption of Support Vector Machine which uses the minimum ranking error as the optimization goal^21^. Similarly, BP-MLL is an extension of the traditional Back-Propagationnetwork^22^ whose cost function was changed to rank loss. IBLR is a KNN-based multi-label learning algorithm which integrates instance-based learning and logistic regression^15^. Classifier Chains (CC) is another way to transform the multi-label learning into traditional single-label classification, which also establishes multiple binary classifiers as BR, but the prediction of the subsequent classifier will be affected by the output of the preceding one, in such a way, the label correlation is considered in the classifier chains. Furthermore, ensemble method (ECC) with different ordered binary classifiers is adopted to reduce the order effect in the chains^23^.

In this study we will propose a novel multi-label learning algorithm, which is composed of two sequential modules. The first module is used to calculate label score for each label respectively, which in fact belongs to BR method. Then the second module will comprehensively consider all label scores and give the final prediction. So the label correlation is considered in a very simple yet effective way in which we neither need to create many new class labels like the LP or RAkEL method, nor need to construct chains of classifiers like CC or ECC, which is very time-consuming. What’s more, the cost function used in our method is also different from the ones used in Rank-SVM or BPMLL. Experiments on the newly constructed AMP dataset will demonstrate the superiority of the proposed method.

## Materials and Methods

### Dataset

The antimicrobial peptide samples were extracted from the APD database, which focused on the natural antimicrobial peptides with defined sequence and activity^4^. In May 2016, there were 2501 samples with APD ID as the identifier, which began with ‘AP’ and five-digit number followed. As we know, the activities of AMPs are not limited to antimicrobial. Among all the AMPs in this dataset, 12 activities (i.e. terminology ‘labels’ for machine learning) were covered. The biological terminology and number of sequences for each activity were listed in Table 1, from which we can see that the most popular activity was Antibacterial that covered about 90% of AMPs, and the Anti-protist was the rarest. The Label Cardinality (LC)^18^ and Label Density(LD)^24^ are used to measure the multi-labeled degree of this dataset. LC is the average number of labels per sample:

$$LC=\frac{1}{2501}\sum _{i=1}^{2501}|{{\boldsymbol{y}}}_{i}|=1.54,$$

where $|{{\boldsymbol{y}}}_{i}|$ is the number of activities (or labels) covered by the *i*th sample. LD is the average number of labels of the examples divided by number of labels: LD = LC/12 = 0.13. The numbers of AMPs with different number of activities were listed in Table 2. About 58% AMPs were relevant to only one activity.

**Table 1:** Number of sequences for different activities.

No.	Activity	Count
1	Antibacterial Peptides (Antibiofilms)	2255
2	Antiviral Peptides (Anti-HIV)	177
3	Antifungal Peptides	988
4	Antiparasitic Peptides (Antimalaria)	84
5	Anticancer Peptides	195
6	Anti-protist Peptides	4
7	Insecticidal Peptides	28
8	Spermicidal Peptides	12
9	Chemotactic peptides	56
10	wound healing	15
11	Antioxidant peptides	19
12	Protease inhibitors	22

**Table 2:** Number and percentage of AMPs with different number of activities.

Number of activities	Number of AMPs	Percentage (%)
1	1449	57.94
2	829	33.15
3	172	6.88
4	34	1.36
5	12	0.48
6	2	0.08
7	1	0.04
8	1	0.04
9	1	0.04
10	0	0
11	0	0
12	0	0
In total	2501	100

**Figure 1:**
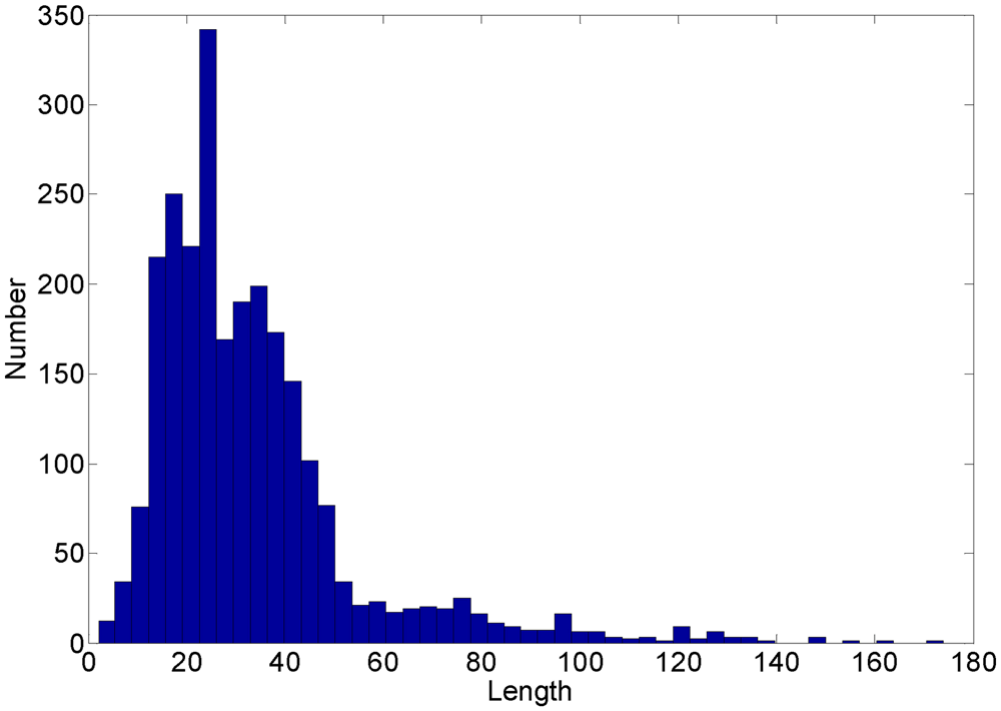
Sequence length distribution in APD.

The sequence length distribution of all AMPs in APD is shown in Fig. 1, from which we can see that most AMPs are 5~60 in length. In this dataset, the shortest sequence is AP02381 consisted of only two amino acid residues; while the longest one is AP02157 with 174 residues.

From Table 1 we can see that the original dataset derived from APD is very unbalanced, so we filtered this dataset by eliminating classes with less than 50 peptides. Additionally, because many small peptides would have AMP activity by chemical modification, and many proteins (>60 residues in length) may be proteolyzed into multiple peptides with multiple activities, so we also eliminated peptides with less than 10 residues or larger than 60 residues. Then there will be 2,222 AMPs and 5 possible activities left and the number of sequences for each activity are listed in Table 3. For the filtered dataset, LC = 1.49 and LD = 0.30.

**Table 3:** Number of sequences for different activities in the filtered dataset.

No.	Activity	Count
1	Antibacterial Peptides (Antibiofilms)	2006
2	Antiviral Peptides (Anti-HIV)	155
3	Antifungal Peptides	903
4	Antiparasitic Peptides (Antimalaria)	70
5	Anticancer Peptides	178

### Feature Extraction

Sequence feature extraction is the foundation of most machine learning methods, including multi-label learning. From some literature^4^ we know that the amino acid composition (AAC) is the most important factor for peptide classification and design, so it may be a good choice. AAC is defined as the 20-dimensional feature vector consists of the frequencies of different amino acids, and it has been successfully used for many protein classification problems^25–27^. Concretely, given an amino acid sequence **P** with *L* in length,

1 $${\bf{P}}={{\rm{A}}}_{{\rm{1}}}{{\rm{A}}}_{{\rm{2}}}{{\rm{A}}}_{{\rm{3}}}{{\rm{A}}}_{{\rm{4}}}{{\rm{A}}}_{{\rm{5}}}{{\rm{A}}}_{{\rm{6}}}{{\rm{\ldots }}A}_{L}$$

where A_1_ is the first amino acid residue in sequence, A_2_ is the second one, and so forth. This sequence can be represented as the AAC vector as [*a*_1_, *a*_2_, …, *a*_20_]^T^, in which *a*_*i*_ (*i* = 1, 2, …, 20) are the occurrence frequencies of the 20 native amino acids, and T is the transpose operator.

**Figure 2:**
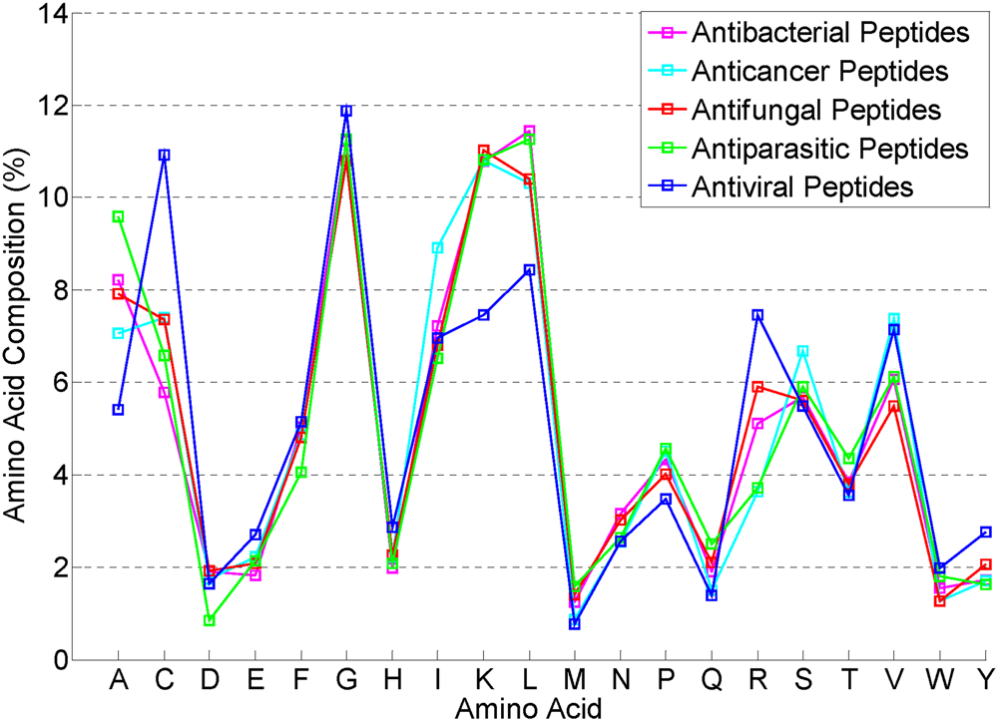
Averaged amino acid composition of AMPs with different activities in the filtered dataset.

The averaged AAC of AMPs with different activities in the filtered dataset are shown in Fig. 2. It seems that AMPs with different activities have different amino acid composition, which is the foundation of functional diversity. Some similar patterns could also be found among different activities. This is not useless because the label correlation may be reflected.

There is a material weakness for representing AMPs as AAC only because no sequence order information is considered. So the dipeptide composition (DC)^28,29^, the frequencies of different dipeptides (combinations of two adjacent amino acids), is also used to represent AMP sequence. The DC is a 400-dimensional vector as [*d*_1_, *d*_2_, …, *d*_400_]^T^, in which *d*_*i*_ (*i* = 1, 2, …, 400) is the occurrence frequency of the *i*th dipeptide. Now any AMP sequence can be converted to a 420-dimensional feature vector as below,

2 $${\boldsymbol{x}}={[{x}_{1},{x}_{2},\ldots ,{x}_{20},{x}_{21},{x}_{22},\ldots ,{x}_{420}]}^{{\rm{T}}}$$

in which the first twenty features are AAC and the other ones are DC.

### Notational Conventions for Multi-label Learning

Let **Ω **⊂ **R**^*d*^ denote a *d*-dimensional sample space, and ${\rm{\Gamma }}=\{{\gamma }_{j}|j=1,...,c\}$ be the finite label set with *c* possible class labels. Each sample ***x*** ∈ **Ω** is associated with a label set ***y ***⊆ **Γ**. In general, the label set ***y*** is represented as a *c*-dimensional binary label vector, in which *y*_*i*_ = +1 means that label γ_*i*_ is relevant to ***x***, while *y*_*i*_ = −1 indicates that γ_*i*_ is irrelevant to ***x***. The goal of multi-learning is to find a function *h*: **Ω → P**(**Γ**), the power set of Γ, from the training dataset $D=\{({{\boldsymbol{x}}}_{i},{{\boldsymbol{y}}}_{i})|i=1,2,\cdots ,n\}$. Then for any unknown sample ***x***, the trained multi-label classifier can estimate its label set *h*(***x***) ⊆ **Γ**. In most cases, the multi-label learning system do not offer the predicted labels directly, but the output values for all labels by a real-valued function *f*: **Ω** × **Γ** → **R**^*c*^, and the output *f*(***x***, *γ*) can be regarded as the confidence of label *γ* is associated with ***x***. Based on these output values, the prediction label set can be obtained by threshold segmentation^30^.

3 $$h({\boldsymbol{x}})=\{\gamma |f({\boldsymbol{x}},\gamma )\ge t,\gamma \in {\rm{\Gamma }}\}$$

where $t\in {\bf{R}}$ is a threshold value. The outputs of relevant labels should be larger than the outputs of irrelevant labels, i.e., $f({\boldsymbol{x}},\gamma ^{\prime} ) > f({\boldsymbol{x}},\gamma ^{\prime\prime} )$ when $\gamma ^{\prime} \in {\boldsymbol{y}}$ and $\gamma ^{\prime\prime} \notin {\boldsymbol{y}}$^16^.

### The Proposed Method

**Figure 3:**
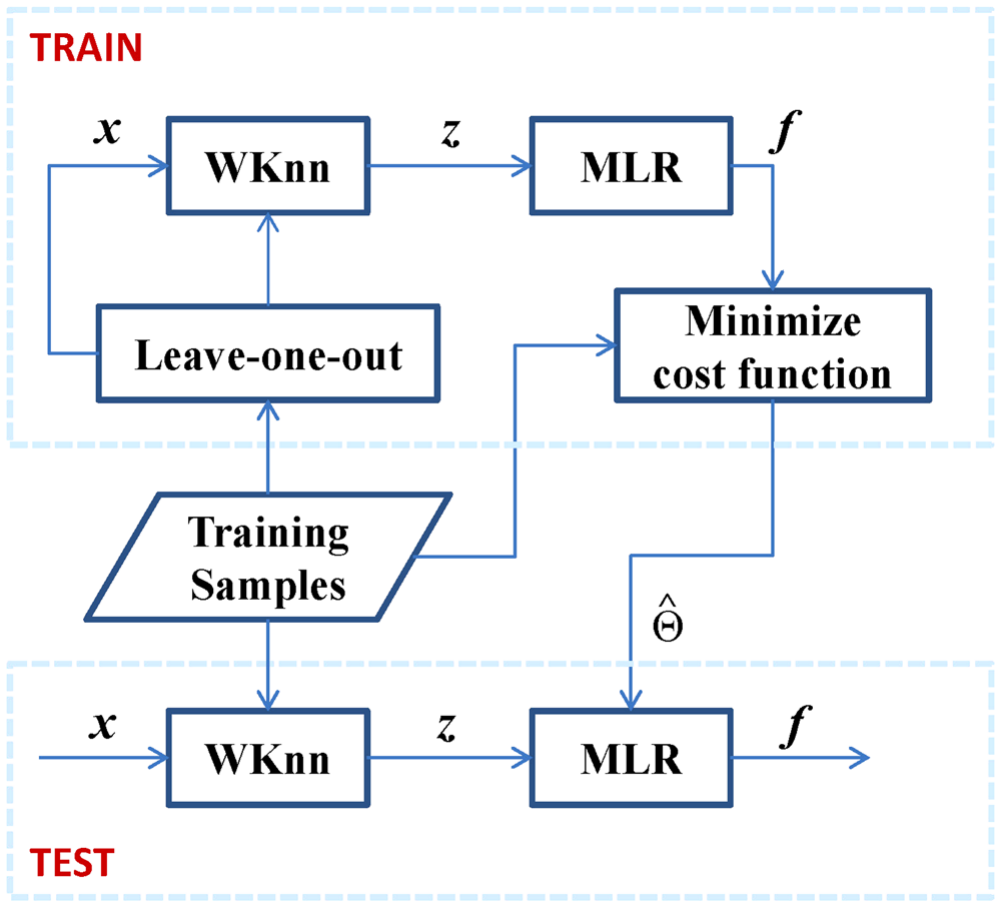
Structure diagram of the proposed multi-label learning method.

There are two serial modules in the proposed method (Fig. 3). The first one is the weighted K nearest neighbor algorithm (WKnn), which is used to get a c-dimensional label score vector for any input sample, then the final outputs of this sample are obtained by the second module-multiple linear regression (MLR), which is the same with the output layer of Extreme Learning Machine(ELM)^31^. In the training procedure, the label scores of the training samples are calculated in Leave-one-out way, which means that for any training sample, the rest is used for neighbor searching. This prevents bias or else the label scores of training samples and testing samples will be very different. When all the label scores pass the MLR model, optimized parameters in MLR are estimated by minimizing a cost function like in Equation (7). After training, the outputs and predicted labels of any query sample will be easily got in the testing procedure. Now let’s give the detail of the two modules.

WKnn is an improvement on the original K nearest neighbor rule. Its basic idea is to weight the evidence of the neighbor according to the similarities with the unknown sample, and the larger the similarity is, the more voting rights the neighbor will have. The similarity of any two samples ***x***_A_ and ***x***_B_ is measured in a max-min method as below

4 $$Sim({{\boldsymbol{x}}}_{{\rm{A}}},{{\boldsymbol{x}}}_{{\rm{B}}})=\frac{\sum _{i=1}^{d}{x}_{{\rm{A}},i}\wedge {x}_{B,i}}{\sum _{i=1}^{d}{x}_{{\rm{A}},i}\vee {x}_{B,i}}$$

where ∧ means taking the small and ∨ taking the large. When an unknown sample ***x*** is to be classified, its *K* nearest neighbors in the training dataset *D* associated with their class labels are given by $({{\boldsymbol{x}}}_{k}^{\ast },{{\boldsymbol{y}}}_{k}^{\ast }),1\le k\le K$. Let the similarities between *x* and these neighbors be ${s}_{k}(1\le k\le K)$ respectively, which have been ordered so that ${s}_{1}\le {s}_{2}\le \cdots \le {s}_{K}$, then the weight of the *k*th nearest neighbor can be defined as the normalized similarities,

5 $${w}_{k}=\{\begin{array}{c}\frac{{s}_{k}-{s}_{1}}{{s}_{K}-{s}_{1}},\,\,\,\,\,\,{s}_{K}\ne {s}_{1}\\ 1,\,\,\,\,\,\,\,\,\,\,\,\,\,\,\,\,{s}_{K}={s}_{1}\end{array}$$

With the weights of neighbors, we can calculate the score of ***x*** for each label as below

6 $${z}_{j}=\sum _{{\gamma }_{j}\in {{\boldsymbol{y}}}_{k}^{\ast }}{w}_{k}/\sum _{k=1}^{K}{w}_{k}\,,\,1\le j\le c$$

Obviously, the more neighbors have the label γ_*j*_, then the larger score may be obtained, and the score varies from a maximum of one when this label is relevant to all the neighbors down to a minimum of zero when no neighbor has this label.

Once any sample is converted to its label score vector, then its final output can be obtained through MLR model. Let ${\rm{Z}}\in {{\bf{R}}}^{n\times (c+1)}$ be the label score matrix with the *i*th row corresponding to the label score vector ***z***_*i*_ of the training sample ***x***_*i*_, which has been augmented so that ${{\boldsymbol{z}}}_{i}={[1{z}_{i,1}{z}_{i,2}\cdots {z}_{i,c}]}^{{\rm{T}}}$. Let ${\rm{Y}}\in {{\bf{R}}}^{n\times c}$ be the label matrix with the *i*th row corresponding to the *c*-dimensional label vector of sample ***x***_*i*_. To minimize the output error, we have the following optimization goal,

7 $$\min \,J({\rm{\Theta }})=\frac{1}{2}{\Vert {\rm{Y}}-{\rm{Z}}{\rm{\Theta }}\Vert }_{F}^{2}+\frac{\lambda }{2}{\Vert {\rm{\Theta }}\Vert }_{F}^{2}$$

where ${\rm{\Theta }}\in {{\bf{R}}}^{(c+1)\times c}$ is the coefficient matrix, ${\Vert \bullet \Vert }_{F}$ indicates the Frobenius norm. On the right-hand side of the equation, the first term is the square of errors, while the second term is the regularization term, which is used to reduce the parameter value and avoid overfitting. The non-negative λ is the tradeoff of the two terms. The regression coefficient matrix Θ can be determined by setting ${\nabla }_{{\rm{\Theta }}}J({\rm{\Theta }})=0$, then we can get the best parameter estimation

8 $$\hat{{\rm{\Theta }}}={({{\rm{Z}}}^{{\rm{T}}}{\rm{Z}}+\lambda {\rm{I}})}^{-1}{{\rm{Z}}}^{{\rm{T}}}{\rm{Y}}$$

For any unknown sample ***x*** whose augmented score vector is ***z***, then the output for all labels can be calculated by

9 $$f({\boldsymbol{x}},{\rm{\Gamma }})={{\boldsymbol{z}}}^{{\rm{T}}}\hat{{\rm{\Theta }}}$$

This procedure is very useful to incorporate the label correlation. For example, the final output *f*(***x***, γ_*i*_) is determined by not only its own label score *z*_*i*_ but also the scores of the other labels *z*_*j*_ (1 ≤ *j* ≤ c, *i* ≠ *j*). In the frame of minimizing the residual sum of squares, if a sample is relevant to one label, then the output of this label will tend to 1. By contrast, if it doesn’t have the label, then the output will tend to −1. The middle value zero is set as the threshold in Equation (3) to separate the relevant labels from the irrelevant labels.

### Performance Measurements

The evaluation criteria on the multi-label dataset are very different from the traditional singe-label dataset because each sample may have one or more class labels. Many complicated evaluation metrics have been proposed specially for multi-label learning in the literature^16,18,19,32^. The following ones are used in this work: (1) Hamming Loss, (2) Subset Accuracy, (3) One Error, (4) Coverage, (5) Ranking Loss, (6) Average Precision, and (7) Micro-averaging F1 (Fmicro). Hamming Loss, Subset Accuracy and Fmicro are label-based metrics, while the others are rank-based ones. It should be noted that a higher value is better for subset accuracy, average precision or Fmicro, but lower is better for the others.

## Results and Discussion

### Influence of Superparameters

**Figure 4:**
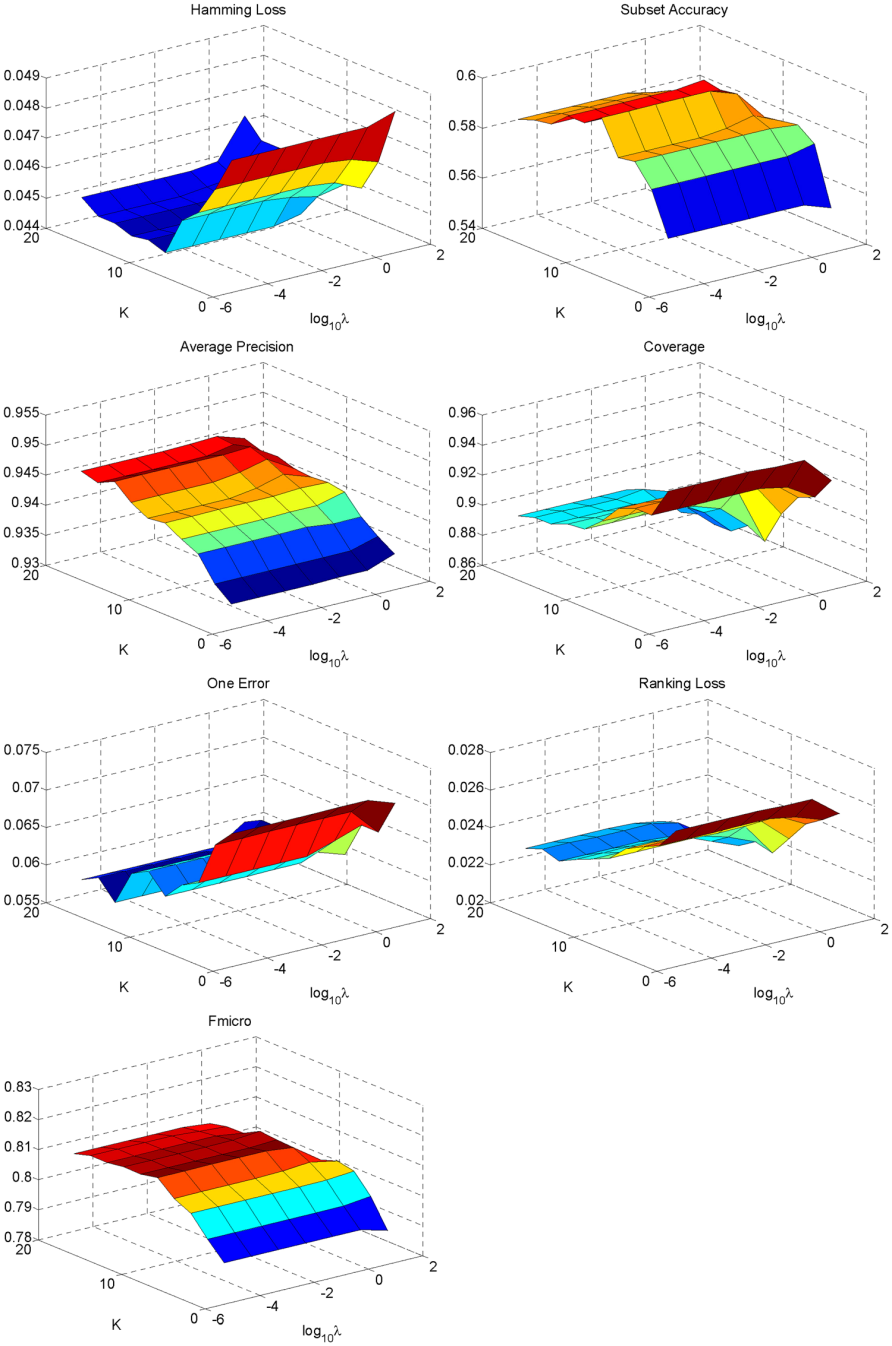
Metric values with different combinations of superparameters by hold-out test on the original dataset. The two horizontal axes represent the values of the two superparameters, and the vertical axis represents the values of the metrics. For clarity the big metric values are mapped in red color while the small values are mapped in blue color.

There are two superparameters in our proposed method, i.e. the number of neighbors *K* and the regularization parameter λ in Equation (7). Hold-out test was carried out to evaluate the influence of the two parameters, in which 1/3 samples were randomly picked out as the testing set and the remainder was for training. The metric values with different combinations of parameters are shown in Fig. 4, from which we can find that the influence of λ is not as much as *K*. With the increasing of *K*, the performance is improved quickly at first, and then reaches a plateau after about *K* = 10. Taking all metrics into account, *K* = 15 and λ = 1 are set as the default.

### Comparison with Different Multi-label Learning Methods

In this section, we compared the proposed method with several existing multi-label learning algorithms, including MLkNN^20^, BPMLL^22^, IBLR^15^, RAkEL, CC and ECC^23^. All the compared methods can be implemented in the Mulan library^33^, which is a Java package for multi-label learning. We grid-searched the parameters of the other methods by cross-validation on the AMP dataset and the best results were used for comparison. In the Mulan library, there is only one parameter for MLkNN and IBLR. The number of neighbors in both methods is determined from the values 2 to 20 with step 2. The default set is used in BPMLL. For the other algorithms, we choose the J48 with default parameters as the base learner then tune the remaining arguments. RAkEL requires two parameters to be tuned. The values 1*c, 2*c, and 3*c (c is the number of possible labels) are considered for the parameter NumberOfModel (number of models), while 3, 6, and 9 are considered for the parameter SizeOfSubset (size of subset). There are no extra arguments for CC but three ones for ECC, i.e. NumOfModels, doUseConfidences (Whether the output is computed based on the average votes or on the average confidences), and doUseSamplingWithReplacement (Whether to use sampling with replacement to create the data of the models of the ensemble). The first parameter is set to 30 and the others are set to ‘true’.

Using the above multi-label learning methods, 10 repeats of 5-folds cross validation (5-CV) are conducted on the original AMP dataset, and the averaged results of seven metrics together with their standard deviations are summarized in Table 4. As can be seen, the averaged results of the proposed method are better than all the others nearly in all the metrics, and its standard deviations are always relatively small. The above evidence strongly suggests that our method is not only effective, but also robust. The performance of BPMLL seems to be the worst. BPMLL is a neural network based model with the rank loss as cost function for multi-label learning, however experiments in^34^ showed that the performance could be improved significantly by changing this cost function, so we also discard the rank loss cost function, and choose one like in Equation (7). MLkNN and IBLR are both KNN-based methods, and their results are very similar. Interestingly, despite the poor performance of CC, its ensemble version ECC is much improved.

**Table 4:** Metric values of different multi-label learning methods through 5-CV on the original dataset.

Method	Proposed	MLkNN	BPMLL	IBLR	RAkEL	CC	ECC
Metric							
Hamming Loss ↓	0.0454 ± 0.0004	0.0528 ± 0.0006	0.2977 ± 0.0227	0.0523 ± 0.0007	0.0540 ± 0.0007	0.0600 ± 0.0015	0.0502 ± 0.0008
Subset Accuracy ↑	0.5988 ± 0.0049	0.5450 ± 0.0040	0.0014 ± 0.0010	0.5494 ± 0.0048	0.5258 ± 0.0069	0.4992 ± 0.0082	0.5662 ± 0.0082
Average Precision ↑	0.9439 ± 0.0011	0.9326 ± 0.0016	0.6691 ± 0.0680	0.9326 ± 0.0013	0.8853 ± 0.0023	0.8474 ± 0.0044	0.9210 ± 0.0020
Coverage ↓	0.9337 ± 0.0104	0.9859 ± 0.0105	2.0595 ± 0.1971	0.9980 ± 0.0084	1.8996 ± 0.0332	2.0774 ± 0.0698	1.3728 ± 0.0207
One Error ↓	0.0607 ± 0.0018	0.0768 ± 0.0026	0.4820 ± 0.1523	0.0752 ± 0.0025	0.1028 ± 0.0029	0.1711 ± 0.0070	0.0756 ± 0.0030
Ranking Loss ↓	0.0234 ± 0.0005	0.0269 ± 0.0006	0.1120 ± 0.0197	0.0275 ± 0.0005	0.0809 ± 0.0026	0.0947 ± 0.0045	0.0473 ± 0.0014
Fmicro ↑	0.8082 ± 0.0015	0.7679 ± 0.0022	0.4437 ± 0.0190	0.7758 ± 0.0025	0.7828 ± 0.0030	0.7574 ± 0.0048	0.7896 ± 0.0035

↓ means lower is better; ↑ means higher is better.

Furthermore, to compare different multi-label learning methods with statistical significance, the paired t-test with the significance level 0.05 was carried out on the 10 repeats of 5-CV results. The comparison triplet CT(A, B) = (win/tie/loss) is used to count the events that algorithm A performs better than algorithm B, the two algorithms perform equally, or algorithm A performs worse than algorithm B. The results of comparison triplets are shown in Table 5, in which each triplet is obtained by the comparison between the algorithm in the row (algorithm A) and the other one in the column (algorithm B). The sum of each triplet is seven, which is the number of metrics to measure the performance of algorithms. The triplets in the last column are the sums of the triplets in each row. Amazingly, the proposed method performed significantly better than all the other ones in all metrics. The second place is taken by the ensemble method ECC, which surpasses nearly all the others except the proposed. IBLR and MLkNN are also KNN-based methods, and the former performs slightly better than the later, but they both fall far behind the proposed method. In terms of the total triplets in the last column of Table 5, all the multi-label learning methods can be ranked as Proposed > ECC > IBLR > MLkNN > RAkEL > CC > BPMLL, where symbol > means ‘better than’. It should be noted that all the comparison is conducted on only the AMP dataset, so we cannot state that the proposed method is better than the other methods in other cases.

**Table 5:** Comparison triplets CT(A, B) = (win/tie/loss) by paired t-test between each pair of methods on the original dataset.

B	Proposed	MLkNN	BPMLL	IBLR	RAkEL	CC	ECC	In total
A								
Proposed	—	7/0/0	7/0/0	7/0/0	7/0/0	7/0/0	7/0/0	42/0/0
MLkNN	0/0/7	—	7/0/0	2/4/1	3/1/3	7/0/0	0/1/6	19/6/17
BPMLL	0/0/7	0/0/7	—	0/0/7	0/0/7	0/1/6	0/0/7	0/1/41
IBLR	0/0/7	1/4/2	7/0/0	—	3/1/3	7/0/0	0/0/7	18/5/19
RAkEL	0/0/7	3/1/3	7/0/0	3/1/3	—	7/0/0	0/1/6	20/3/19
CC	0/0/7	0/0/7	6/1/0	0/0/7	0/0/7	—	0/0/7	6/1/35
ECC	0/0/7	6/1/0	7/0/0	7/0/0	6/1/0	7/0/0	—	33/2/7

It is meaningful to compare the computational efficiency. We tested all the algorithms with default parameters by 5-fold cross-validation on a computer with the Intel Core i3 CPU @3.4 GHz and 4 GB memory. In Java IDE, the single-run execution time of the algorithms MLKNN, IBLR, BPMLL, RAKEL, CC and ECC were 98.67, 107.05, 148.46, 583.49, 149.01 and 3248.32 in seconds, respectively. While in MATLAB IDE the proposed method ran for 205.20 seconds. Prospectively the Java version of the proposed method will be more efficient. So the running time of the proposed is reasonably well.

It should be noted that some AMPs with very short length in the original dataset are antimicrobial because of chemical modification, and the learning or predicting based on amino acid composition lacks biological meaning, so it is preferable to construct multi-label learning models using the filtered dataset.

When conducting Hold-out test on the filtered dataset with the proposed method, the metric landscapes in regard to the hyperparameters were very similar to Fig. 4. Therefore, K = 15 and λ = 1 are also set as the default parameters. For the other algorithms, all the parameters were grid-searched like the procedure above and the best results were chosen for comparison. Similarly, 10 repeats of 5-CV are conducted on the filtered dataset, and the averaged results of seven metrics together with their standard deviations are listed in Table 6. With the cross-validation results, the paired t-test with the significance level 0.05 is carried out and the results of comparison triplets are shown in Table 7. From Tables 6 and 7 we can find that the proposed method is still the best one.

**Table 6:** Metric values of different multi-label learning methods through 5-CV on the filtered dataset.

Method	Proposed	MLkNN	BPMLL	IBLR	RAkEL	CC	ECC
Metric							
Hamming Loss ↓	0.0992 ± 0.0014	0.1083 ± 0.0009	0.6366 ± 0.0214	0.1073 ± 0.0007	0.1139 ± 0.0023	0.1258 ± 0.0025	0.1055 ± 0.0007
Subset Accuracy ↑	0.6141 ± 0.0056	0.5874 ± 0.0033	0.0022 ± 0.0008	0.5901 ± 0.0040	0.5594 ± 0.0065	0.5280 ± 0.0108	0.5928 ± 0.0035
Average Precision ↑	0.9553 ± 0.0010	0.9501 ± 0.0008	0.4018 ± 0.0416	0.9506 ± 0.0011	0.9289 ± 0.0022	0.8821 ± 0.0049	0.9505 ± 0.0009
Coverage ↓	0.6899 ± 0.0054	0.7050 ± 0.0038	2.7546 ± 0.2624	0.7006 ± 0.0034	0.8208 ± 0.0108	1.0545 ± 0.0253	0.7032 ± 0.0051
One Error ↓	0.0565 ± 0.0021	0.0669 ± 0.0012	0.9224 ± 0.0562	0.0670 ± 0.0020	0.0888 ± 0.0037	0.1517 ± 0.0085	0.0661 ± 0.0019
Ranking Loss ↓	0.0444 ± 0.0010	0.0481 ± 0.0006	0.6203 ± 0.0829	0.0471 ± 0.0008	0.0714 ± 0.0025	0.1223 ± 0.0053	0.0473 ± 0.0010
Fmicro ↑	0.8226 ± 0.0026	0.8011 ± 0.0020	0.4509 ± 0.0135	0.8050 ± 0.0014	0.8064 ± 0.0037	0.7834 ± 0.0038	0.8131 ± 0.0011

↓ means lower is better; ↑ means higher is better.

**Table 7:** Comparison triplets CT(A, B) = (win/tie/loss) by paired t-test between each pair of methods on the filtered dataset.

B	Proposed	MLkNN	BPMLL	IBLR	RAkEL	CC	ECC	In total
A								
Proposed	—	7/0/0	7/0/0	7/0/0	7/0/0	7/0/0	7/0/0	42/0/0
MLkNN	0/0/7	—	7/0/0	0/3/4	6/0/1	7/0/0	0/4/3	20/7/15
BPMLL	0/0/7	0/0/7	—	0/0/7	0/0/7	0/0/7	0/0/7	0/0/42
IBLR	0/0/7	4/3/0	7/0/0	—	6/1/0	7/0/0	0/5/2	24/9/9
RAkEL	0/0/7	1/0/6	7/0/0	0/1/6	—	7/0/0	0/0/7	15/1/26
CC	0/0/7	0/0/7	7/0/0	0/0/7	0/0/7	—	0/0/7	7/0/35
ECC	0/0/7	3/4/0	7/0/0	2/5/0	7/0/0	7/0/0	—	26/9/7

### Comparison with iAMP-2L

iAMP-2L is a two-level classifier for AMPs, in which the first level is used to identify a peptide sequence as AMP or not, if it is, then its activities will be predicted in the second level^13^. Because the proposition of the current work focuses on multi-label learning, only the method used in the second level of iAMP-2L is picked out for comparison. In fairness, two multi-label learning algorithms with two different feature extraction methods are all tested by 10 runs of 5-CV on the originally constructed dataset, so there are four sets of experiment results listed in Table 8, in which the best values of different metrics are bolded. Obviously, the feature extraction method in Equation (2) and the novel multi-label learning algorithm are the winning combination. With the same feature extraction, the proposed multi-label learning algorithm significantly outperforms the one used in iAMP-2L. With the same multi-label learning algorithm, the feature extraction method used in this work is slightly better than the pseudo amino acid composition (PseAAC)^35,36^. Perhaps this is because the minimum sequence length in the new dataset is two and only the first-order correlation factors could be extracted, so the power of PseAAC is restrained.

**Table 8:** The means and standard deviations of 5-CV results with the proposed method and iAMP-2L when testing on the original dataset.

Method	Proposed^a^	Proposed^b^	iAMP-2L^a^	iAMP-2L^b^
Metric				
Hamming Loss ↓	**0.0454 ± 0.0004**	0.0483 ± 0.0005	0.0580 ± 0.0003	0.0581 ± 0.0007
Subset Accuracy ↑	**0.5988 ± 0.0049**	0.5733 ± 0.0040	0.4880 ± 0.0041	0.4848 ± 0.0043
Average Precision ↑	**0.9439 ± 0.0011**	0.9383 ± 0.0012	0.9361 ± 0.0010	0.9353 ± 0.0015
Coverage ↓	**0.9337 ± 0.0104**	0.9816 ± 0.0096	1.1006 ± 0.0116	1.1121 ± 0.0161
OneError ↓	**0.0607 ± 0.0018**	0.0689 ± 0.0018	0.0658 ± 0.0013	0.0682 ± 0.0025
Ranking Loss ↓	**0.0234 ± 0.0005**	0.0259 ± 0.0005	0.0385 ± 0.0006	0.0400 ± 0.0010
Fmicro ↑	**0.8082 ± 0.0015**	0.7955 ± 0.0021	0.7852 ± 0.0010	0.7851 ± 0.0026

The superscript a indicates the feature extraction method in this work, and b indicates the PseAAC. ↓ means lower is better; ↑ means higher is better. The best value for each metric is in bold.

Because the results obtained from the original dataset lacks biological significance, we also compare the proposed method and the second level classifier in iAMP-2L on the filtered dataset, in which the minimum sequence length is ten, and higher-order correlation factors can be used. As did in iAMP-2L, five physical-chemical properties are used to code the peptide sequences, while the order of correlation factors are grid-searched from 2 to 8 with step 2, and the four-order is found to be the best choice. So each peptide sequence is converted to a 40-dimensional feature vector. We perform ten runs of 5-CV on the filtered dataset using two different multi-label learning algorithms with two modes of feature extraction method respectively and list the results in Table 9. As a whole, the proposed multi-label learning method is better than the one used in iAMP-2L. The performance of the feature extraction method used in this work is slightly better than PseAAC when using the proposed learning method, whereas the opposite is true for iAMP-2L. The dimension of PseAAC is much lower, and if more appropriate physical-chemical properties or chemical modification information can be incorporated, we believe it will improve its performance, yet this has to be tested in the future.

**Table 9:** The means and standard deviations of 5-CV results with the proposed method and iAMP-2L when testing on the filtered dataset.

Method	Proposed^a^	Proposed^b^	iAMP-2L^a^	iAMP-2L^b^
Metric				
Hamming Loss ↓	**0.0992 ± 0.0014**	0.1018 ± 0.0012	0.1221 ± 0.0020	0.1212 ± 0.0023
Subset Accuracy ↑	**0.6141 ± 0.0056**	0.6033 ± 0.0041	0.5149 ± 0.0063	0.5228 ± 0.0078
Average Precision ↑	**0.9553 ± 0.0010**	0.9534 ± 0.0010	0.9526 ± 0.0014	0.9527 ± 0.0016
Coverage ↓	**0.6899 ± 0.0054**	0.6946 ± 0.0034	0.6911 ± 0.0055	0.6953 ± 0.0064
OneError ↓	**0.0565 ± 0.0021**	0.0615 ± 0.0019	0.0652 ± 0.0022	0.0638 ± 0.0028
Ranking Loss ↓	**0.0444 ± 0.0010**	0.0457 ± 0.0007	0.0494 ± 0.0014	0.0498 ± 0.0015
Fmicro ↑	**0.8226 ± 0.0026**	0.8176 ± 0.0023	0.8083 ± 0.0028	0.8091 ± 0.0033

The superscript a indicates the feature extraction method in this work, and b indicates the PseAAC. ↓ means lower is better; ↑ means higher is better. The best value for each metric is in bold.

## Conclusion

There have been many bioinformatics tools with good ability proposed for identifying a peptide sequence as AMP or not, some of them can obtain the testing accuracy of more than 90%^6,8,12,13^. When we get a peptide with high antimicrobial potential by these tools, then we want to know its specific activities, yet there is few research about the activity prediction of AMPs from the point of multi-label learning. In this work, a new AMP dataset and its filtered version are created. After a detailed analysis of the sequence and activity information, the amino acid composition and dipeptide composition are extracted to represent any AMP sequence as a feature vector. Then several multi-label learning algorithms are tested on the newly constructed datasets. As far as we know, this is the first time to evaluate so many multi-label learning methods for AMP activities prediction. What’s more, a novel multi-label learning method is proposed, in which the label correlation could be taken into account effectively. Results by cross-validation show that the proposed method outperforms the others significantly. At last, we compare the methods used in this work with the ones in iAMP-2L, including feature extraction and multi-label learning algorithm. Experiments show that the newly proposed method is competent for the prediction of AMP activities.
