# Author Correction: Multi-label Learning for Predicting the Activities of Antimicrobial Peptides

**DOI:** 10.1038/s41598-018-28146-x

**Published:** 2018-06-22

**Authors:** Pu Wang, Ruiquan Ge, Liming Liu, Xuan Xiao, Ye Li, Yunpeng Cai

**Affiliations:** 1 Shenzhen Institutes of Advanced Technology, and Key Lab for Health Informatics, Chinese Academy of Sciences, Shenzhen, Guangdong 518055 China; 2 Shenzhen College of Advanced Technology, University of Chinese Academy of Sciences, Shenzhen, 518055 China; 3 Computer Department, Jingdezhen Ceramic Institute, Jingdezhen, 333403 China; 4 College of Mathematics and Statistics, Shenzhen University, Shenzhen, 518060 China; 5 Present Address: School of Computer Science and Technology, Hangzhou Dianzi University, Hangzhou, Zhejiang 310018 China

Correction to: *Scientific Reports* 10.1038/s41598-017-01986-9, published online 19 May 2017

In the original version of this Article, there were errors in Affiliation 5 which was incorrectly listed as ‘Present address: School of Computer Science and Technology, Hanzhou Dianzi University, Hangzhou, Zhejiang, 310018, China.’

The correct affiliation is listed below:

Present address: School of Computer Science and Technology, Hangzhou Dianzi University, Hangzhou, Zhejiang, 310018 China.

These errors have now been corrected in the PDF and HTML versions of the Article.

